# Morphological and hemispheric and sex differences of the anterior ascending ramus and the horizontal ascending ramus of the lateral sulcus

**DOI:** 10.1007/s00429-022-02482-1

**Published:** 2022-04-20

**Authors:** Yu Wang, Feifei Xu, Wenjuan Zhou, Lanwei Hou, Yuchun Tang, Shuwei Liu

**Affiliations:** 1 Department of Anatomy and Neurobiology, Research Center for Sectional and Imaging Anatomy, Shandong Provincial Key Laboratory of Mental Disorder, Shandong Key Laboratory of Digital Human and Clinical Anatomy, School of Basic Medical Sciences, Cheeloo College of Medicine, Shandong University, Jinan, 250012 Shandong China; 2 Institute of Brain and Brain-Inspired Science, Shandong University, Jinan, 250012 Shandong China

**Keywords:** Morphological pattern, Broca’s area, Anterior ascending ramus of the lateral sulcus (aals), Horizontal ascending ramus of the lateral sulcus (hals)

## Abstract

Broca’s area is composed of the pars opercularis (PO) and the pars triangularis (PTR) of the inferior frontal gyrus; the anterior ascending ramus of the lateral sulcus (aals) separates the PO from the PTR, and the horizontal ascending ramus of the lateral sulcus (hals) separates the PTR from the pars orbitalis. The morphometry of these two sulci maybe has potential effects on the various functions of Broca’s area. Exploring the morphological variations, hemispheric differences and sex differences of these two sulci contributed to a better localization of Broca's area. BrainVISA was used to reconstruct and parameterize these two sulci based on data from 3D MR images of 90 healthy right-handed subjects. The 3D anatomic morphologies of these two sulci were investigated using 4 sulcal parameters: average depth (AD), average width (AW), outer length (OL) and inner length (IL). The aals and hals could be identified in 98.89% and 98.33%, respectively, of the hemispheres evaluated. The morphological patterns of these two sulci were categorized into four typical types. There were no statistically significant interhemispheric or sex differences in the frequency of the morphological patterns. There was statistically significant interhemispheric difference in the IL of the aals. Significant sex differences were found in the AD and the IL of the aals and OL of the hals. Our results not only provide a structural basis for functional studies related to Broca’s area but also are helpful in determining the precise position of Broca’s area in neurosurgery.

## Introduction

**Fig. 1:**
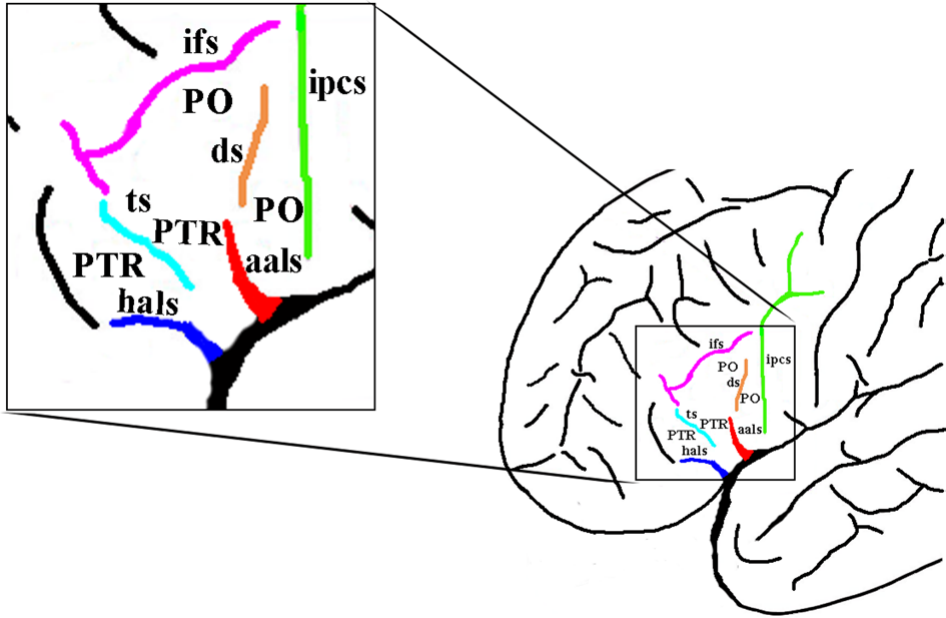
Sulcal maps of the lateral frontal lobe surface. The inferior frontal gyrus has been outlined in black box and expanded on the left. Anterior ascending ramus of the lateral sulcus (aals), the red sulcus; horizontal ascending ramus of the lateral sulcus (hals), the blue sulcus; inferior precentral sulcus (ipcs), the green sulcus; sulcus diagonalis (ds), the yellow sulcus; inferior frontal sulcus (ifs), the purple sulcus; triangularis sulcus, the baby blue; PO, pars opercularis; PTR, pars triangularis

Broca’s area, located in the inferior frontal gyrus of the language dominant hemisphere and above the lateral sulcus, is primarily comprised of the pars triangularis (PTR) and the pars opercularis (PO) (Fig. 1). Impairment to the posterior ventrolateral region of the left hemisphere could lead to expressive aphasia (Broca 1861). Broca's area is traditionally known to be a core region for speech production. A wealth of functional neuroimaging studies has shown that neural activity in the PO and the PTR increases during speech tasks (Petrides et al. 1993; Amunts et al. 2004; Heim et al. 2008; Wu et al. 2012; Clos et al. 2013). In addition, the PTR and the PO are critical for the production of speech, which demonstrated by electrical stimulation (Schaffler et al. 1993; Havas et al. 2015) and lesion studies (Alexander et al. 1990).

Recent research in Broca’s area has mainly focused on the study of Occidental populations, but differences in genetics, culture and environmental exposures may give rise to differences in brain structure and function between Oriental and Occidental populations (Han and Ma 2014; Lou et al. 2019). According to surface-based morphometry (SBM) and voxel-based morphometry (VBM) analysis, compared to Westerners, the Chinese population had smaller structural measures, e.g., cortical thickness, cortical volume in the frontal cortices (Tang et al. 2018). Numerous studies have shown that cultural differences affect the linguistic structure and function of the brain (Ge et al. 2015; Xu et al. 2017). The study of localized morphological brain differences between English-speaking Caucasians (ECs) and Chinese-speaking Asians (CAs) found that the left middle frontal gyrus (LMFG) was larger in CAs than in ECs. In addition, research on developmental dyslexia has found that alphabetic (e.g., English) and non-alphabetic (e.g., Chinese) languages have different structural and functional bases. Specifically, for readers of alphabetic writing systems, impaired reading was associated with dysfunction of the left temporoparietal and occipitotemporal region (Aylward et al. 2003; Johansson 2006; Sun et al. 2010). Conversely, readers of logographic writing systems exhibited reduced gray matter volume in the LMFG, which was previously shown to be important for Chinese reading and writing (Siok et al. 2004, 2008). Therefore, it is necessary to utilize Chinese data to study Broca’s area.

The inferior frontal gyrus consists of area 44, area 45 and area 47/12 with cytoarchitectonic criteria. Area 44 is located in pars opercularis. Area 45 corresponds to the pars triangularis and area 47/12, a subdivision of Brodmann’s original area 47, refers to the pars orbitalis (Petrides and Pandya 2002). The PO is in an ideal region to investigate as it has advanced control of the orofacial musculature, which might relate to the integration of meaningful information (Petrides 2016). Evidence has indicated that Brodmann area (BA) 45 is active in controlled processes at the word level, such as semantic judgement or categorization, lexical-semantic access, and aspects of sentence-level semantic plausibility (Petrides 2014; Westphal et al. 2016). Functional imaging studies of normal human subjects indicate that BA 44 is involved in syntax processing, whereas BA 45 mainly contributes to semantic processing (Goucha and Friederici 2015). The desire to understand the functions of Broca’s area has prompted anatomical studies of the asymmetry of this region. The anterior ascending ramus of the lateral sulcus (aals) and the horizontal ascending ramus of the lateral sulcus (hals) are the important landmarks within Broca’s area. The shape and length of these two sulci vary greatly between individuals, as leads to individual difference in the size, surface area and volume of the PO and PTR. However, there are few studies on the aals and hals, especially in Chinese populations.

Sex differences in brain structure have been reported, ranging from global differences to local differences in regional tissue volume and the size of the substructure (Xu et al. 2000; Luders et al. 2013). Kurth et al. (2017) found that the bilateral gray matter volume of BA 44 and BA 45 was significantly greater in females than in males, while the sex difference was not significant in BA 44/45 asymmetry (Kurth et al. 2017). The sulcal width and depth of men have been reported to change more rapidly with age, compared to these changes in women, especially in the temporal collateral and cingulate sulci (Kochunov et al. 2005). Since only one study has been conducted on sex differences of these two sulci (Powell et al. 2012), further investigation is necessary.

To understand the relationship between the structures of the aals and hals and the function of Broca’s area, in this study, we used a large sample of in vivo brain MRI images and advanced brain imaging and brain structure analysis methods. The present investigation characterized the morphology of the aals and hals to explore hemispheric and sex differences in various morphometric parameters of these two sulci. This study is expected to provide a method for complex brain sulcus analysis, present an anatomical basis for functional and related studies of Broca’s area, and offer basic guidance for functional neuroimaging studies and neurosurgical operations in related brain areas.

## Materials and methods

### Subjects

Ninety Chinese volunteers (mean age 17.30 ± 1.58 years, 50 males and 40 females) were recruited for the study. All subjects had no history of neurological and/or psychiatric illness, corresponding to the Diagnostic and Statistical Manual of Mental Disorders (DSM-IV). All subjects were right handed as determined by the Edinburgh Handedness Inventory. Ethics approval was obtained from the Ethics Committee of Shandong University before the initiation of this study. All participants and their parents provided written informed consent.

### MRI acquisition and processing

High-quality three-dimensional structural MR data were acquired using a 3.0 T GE (General Electric, Milwaukee, USA) MRI Scanner with a standard eight-channel head coil. Acquisition parameters for T1-weighted structural MRI scans were identical and as follows: repetition time (TR) = 18 ms, echo time (TE) = 10 ms, voxel size = 0.47 mm × 0.47 mm × 0.70 mm, field of view (FOV) = 24.0 cm × 24.0 cm, matrix size = 512 × 512, flip angle = 10°, slice thickness = 1.4 mm, slice gap = − 0.7 mm, number of excitations (NEX) = 2, and total scan time = 12 min. All images were scanned along a horizontal line through the anterior and posterior commissures.

### Image processing

**Fig. 2:**
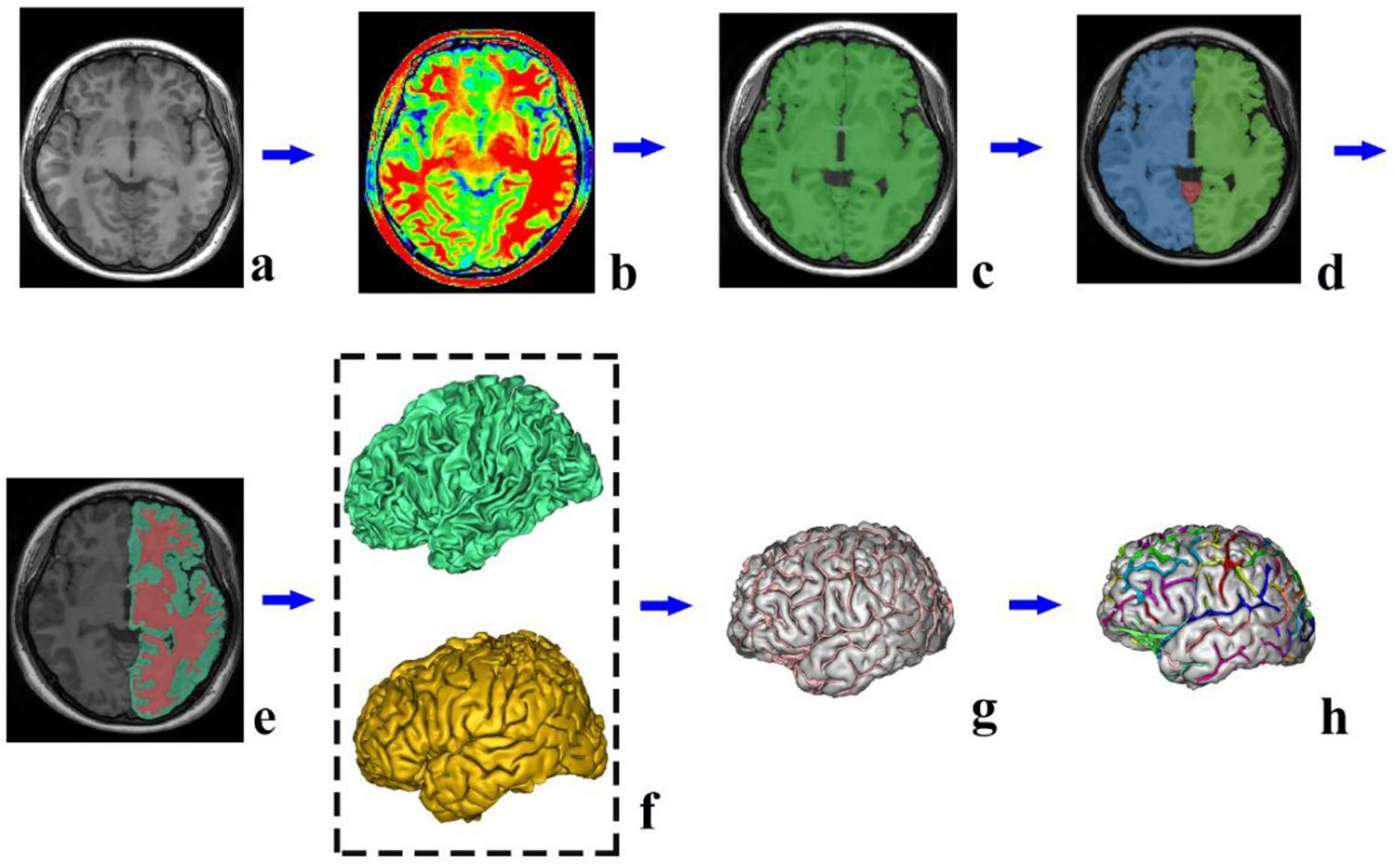
Sulcal extraction and identification pipeline consists of (**a**) import the T1-weighted MRI into BrainVISA database; (**b**) bias correction; (**c**) calculate the mean whole-brain mask; (**d**) meshed the cerebral hemispheres; (**e**) remove the non-brain tissue and segment the brain tissues into GM, WM and CSF; (**f**) reconstruction of white/gray matter mesh; (**g**) sulci recognition and (**h**) automated labeling of sulci

First, all MR images were transformed in FreeSurfer software (http://surfer.nmr.mgh.harvard.edu/fswiki/) to the MNI305 coordinate system to remove gross differences in brain size and orientation using linear registration. Then, the cortical sulci of subjects were reconstructed and automatically identified through BrainVISA (BV) software (http://brainvisa.info/). Particularly, cortical sulci were analyzed using the following steps: import of T1-weighted MRI data, bias correction, calculation of the mean whole-brain mask, meshing of the cerebral hemispheres, removal of non-brain tissue, segmentation of brain tissues into white/gray matter (W/GM) and cerebrospinal fluid (CSF), reconstruction of white/gray matter mesh, sulci recognition and automated labeling of sulci (Liu et al. 2010) (Fig. 2). The labeling was performed automatically, and then the automated result was checked visually by an experienced neuroanatomist. If the automatically labeled sulci name was found to be wrong, we modified it manually. To validate the sulcal morphology observed with BrainVISA, we manually labelled the aals and the hals in each MRI volume using ITK-SNAP (http://www.itksnap.org/pmwiki/pmwiki.php). Each of the individual MRI volume was transformed in FreeSurfer software to the MNI305 coordinate system. The sulci of interest were examined in 1 mm steps, and subsequently, all voxels making up each sulcus were marked in ITK-SNAP. The manual tracking of the aals and hals was mainly completed in the sagittal plane, while the results were corrected in the coronal plane and transverse plane.

### Morphological patterns

The anterior rami of the lateral sulcus, namely, the aals and the hals, were identified based on criteria established in previous studies (Eberstaller 1890; Economo and Koskinas 1925; Petrides 2012). The aals, which marks the division between the PO and PTR, is a deep vertical ramus extending dorsally from the lateral sulcus into the inferior frontal gyrus. The hals delineates the PTR from the pars orbitalis and seems to be an extension of the lateral sulcus in the ventrolateral frontal cortex (Fig. 1). The most important identifying characteristic of the anterior rami of the lateral sulcus is the medial extension to the level of the insula such that the aals and the hals fuse with the circular sulcus of the insula. Such criteria were used for the manual labeling in volume format and for the verification of the automated labeling in BrainVISA. Following this, to classify morphological patterns, BrainVISA was used to navigate through the intrasulcal anatomy in three dimensions to distinguish these two sulci from their neighbors. According to the appearance and superficial connection of the two sulci, they were classified into different morphological types. A similar approach was used when examining the sulcal patterns in the MRI volumes.

### Computation of sulci parameters

**Fig. 3:**
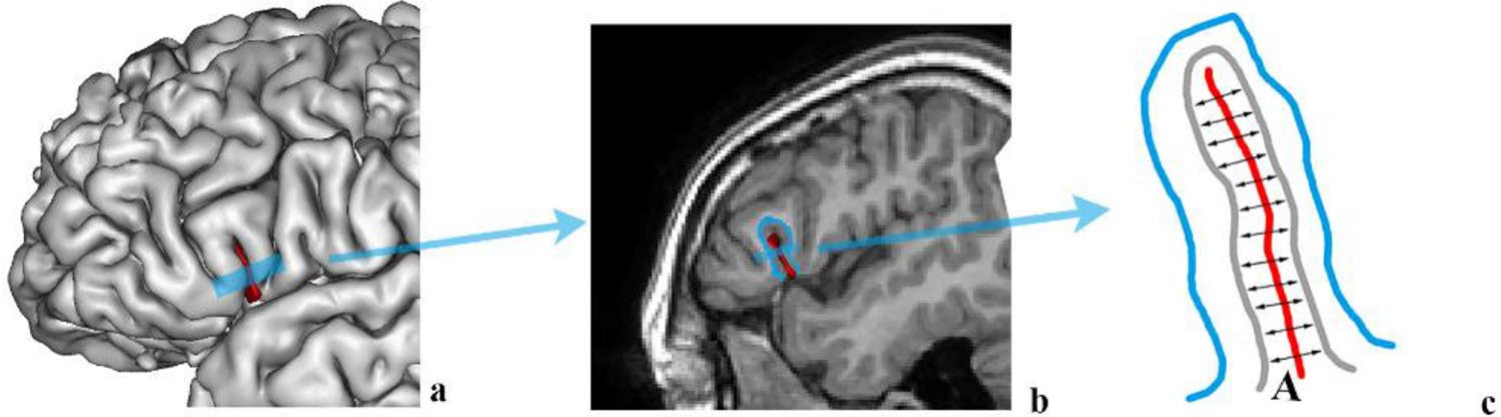
The average sulcal width for an individual sulcal structure is defined as an average 3D distance between opposing points on the GM mesh along the normal projections to the sulcal surface. **a** snapshot of cortical surface extraction, **b** the sagittal section. In the schematic drawing on **c** red line indicates sulcal surface, gray line indicates GM mesh, blue line indicates WM mesh, A indicates sulcal width

**Fig. 4:**
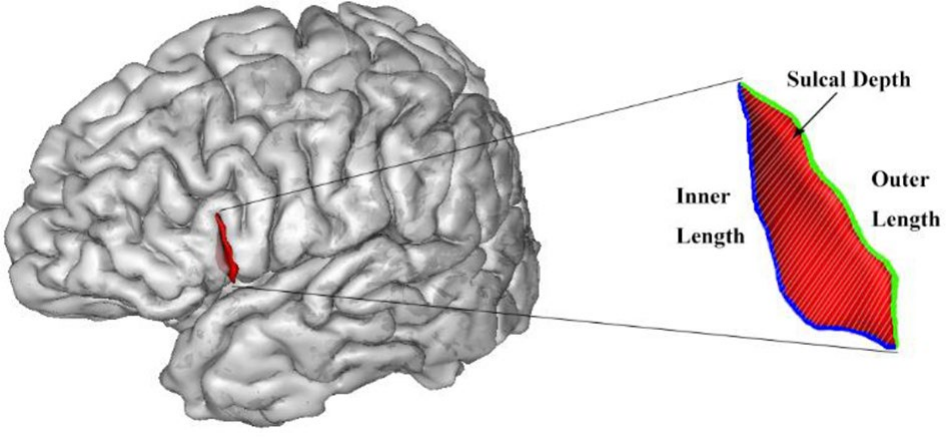
Average sulcal depth is calculated as the Euclidean distance between outer and inner lengths of the sulcal surface. The outer length was defined as the length of the lateral ridge of the sulcus, the outer length is marked green. The inner length was defined as the length of the fundus of the sulcus, the inner length is marked blue. The sulcus is the aals

Measurements of sulcal length, sulcal width, and sulcal depth required RIC tools for BrainVISA (http://ric.uthscsa.edu/personalpages/petr/). The average sulcal width (AW) for an individual sulcal structure was defined as an average 3D span along the normal projections to the medial sulcal mesh (Kochunov et al. 2005) (Fig. 3). Geometrically, a medial sulcal mesh traverses the sulcal space in the middle of the sulcal “span” dimension, parallel to gyral gray matter borders and spans the entire sulcal “depth” dimension. The sulcal width was calculated as the Euclidean distance between two points on the gyral gray matter mesh on either side of the sulcal surface. The sulcal length consists of the outer length (OL) and inner length (IL). The OL was defined as the length of the lateral edge of the sulcus. The IL was defined as the length of the ridge at the base of the sulcus. The average sulcal depth (AD) was the Euclidean distance between the outer and inner lengths of the sulcal surface (Kochunov et al. 2005, 2008) (Fig. 4).

### Statistical analysis

The chi-squared test of independence was conducted to investigate the significance of interhemispheric and sex differences in the frequency of morphological patterns of the aals and hals. Two-way ANOVA with sex as between subject factor and hemisphere as within subject factor was used for analyzing sulcal parameters. *P* < 0.05 was considered statistically significant. Statistical analysis was conducted with the statistical software package SPSS (IBM SPSS, version 25).

## Results

### Morphological patterns of the aals and the hals

**Fig. 5:**
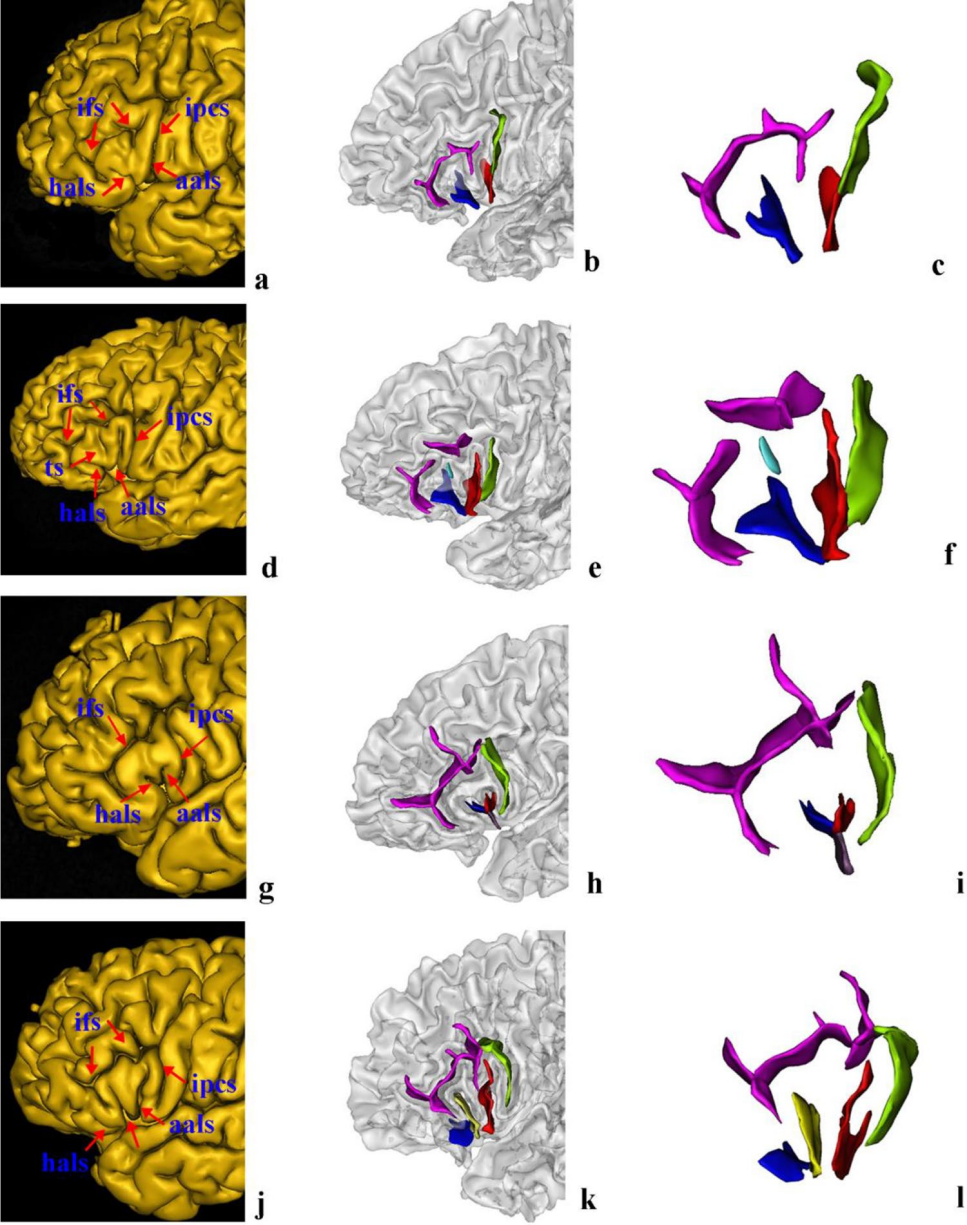
Examples of morphological patterns formed by the anterior ascending ramus of the lateral sulcus (aals) and the horizontal ascending ramus of the lateral sulcus (hals). Each example is illustrated by snapshot of surface extraction and sulci detail. Aals, the red sulci; hals, the blue sulci; the third branch of the lateral sulcus, the pale-yellow sulci; inferior precentral sulcus (ipcs), the green sulci; the inferior frontal sulcus (ifs), the purple sulci; the triangularis sulcus, the baby blue. **a**–**c** Type I was that the aals is clearly separated from the hals, forming the shape of the letter U; **d**–**f**: Type II was that the aals and the hals sprang from a common point, forming the shape of the letter V ; **g**–**i**: Type III was that the aals shares a common stem with hals, forming the shape of the letter Y, the common stem of the aals and hals has been labeled with brown; **j**, **k**, **l**: Type IV was a third anterior ramus of the lateral sulcus between the aals and the hals

**Fig. 6:**
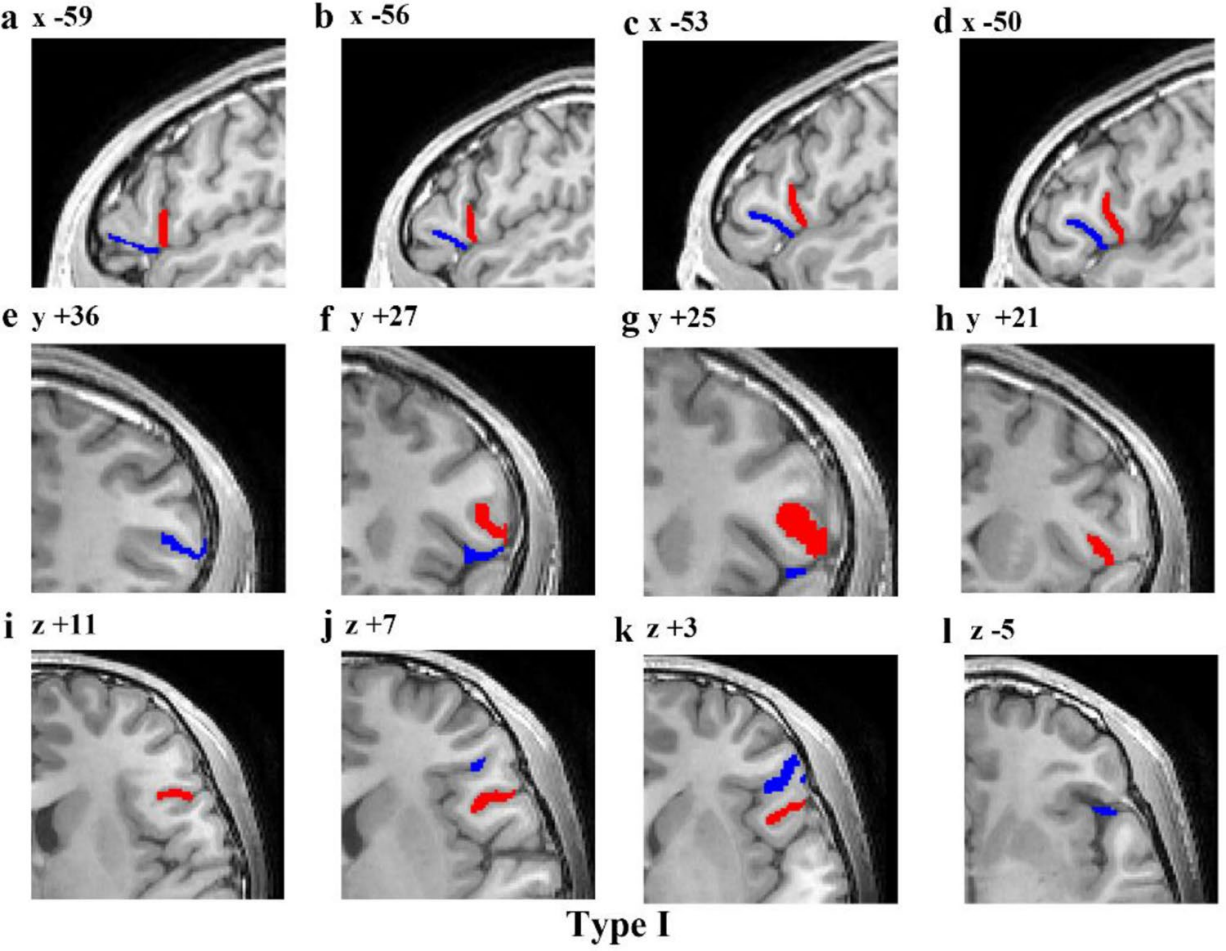
Example of an MRI scan (left hemisphere) illustrating the Type $\mathrm{I}$ morphology pattern. The first line is a series of sagittal sections (lateral to medial), with x coordinates indicated at the top of each section. The second line is a series of coronal sections (anterior to posterior), with *y* coordinates indicated at the top of each section. And the third line is a series of transverse sections (dorsal to ventral), with *z* coordinates indicated at the top of each section. Red represents the aals, blue represents the hals

**Fig. 7:**
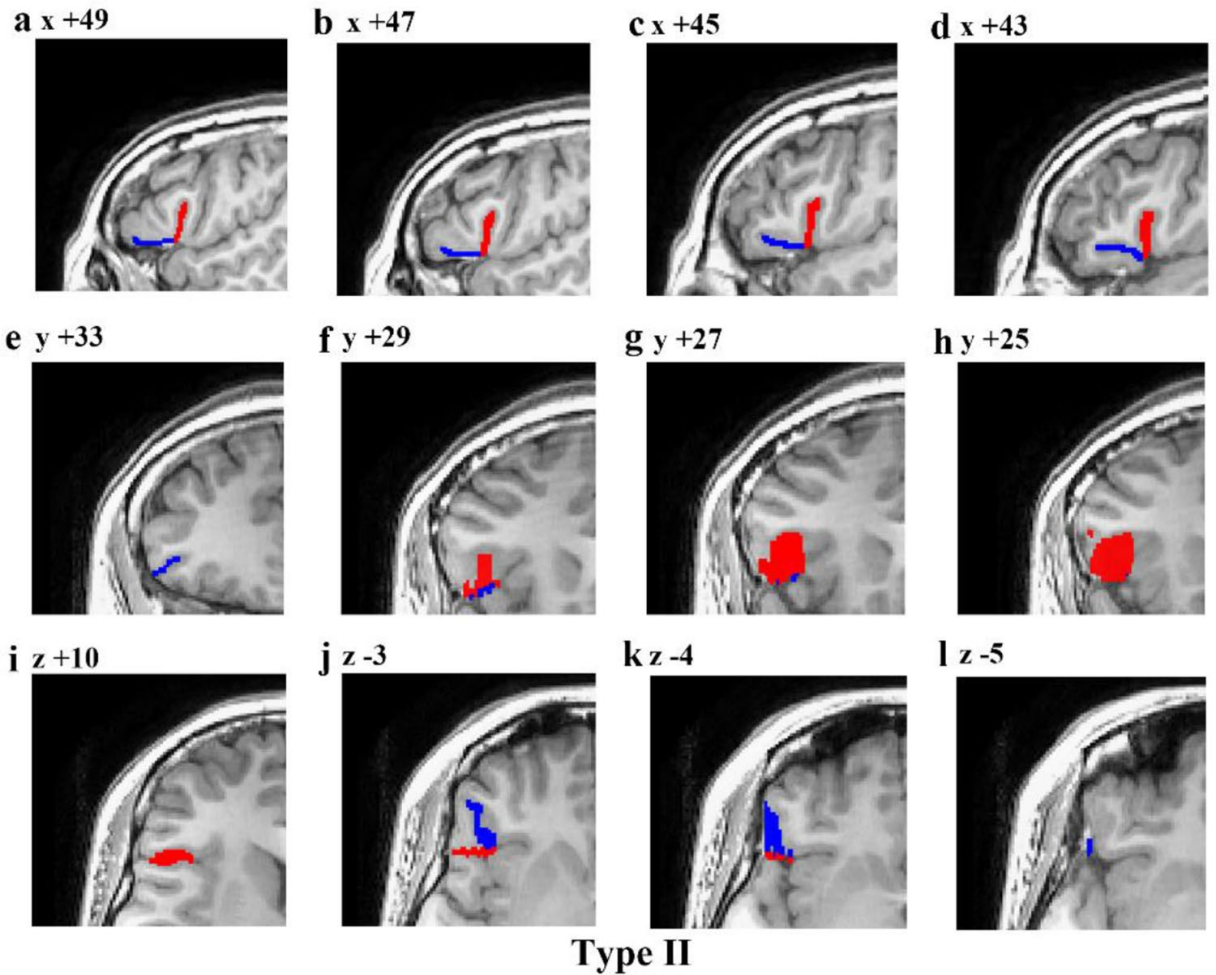
Example of an MRI scan (right hemisphere) illustrating the Type II morphology pattern. The first line is a series of sagittal sections (lateral to medial), with x coordinates indicated at the top of each section. The second line is a series of coronal sections (anterior to posterior), with *y* coordinates indicated at the top of each section. And the third line is a series of transverse sections (dorsal to ventral), with *z* coordinates indicated at the top of each section. Red represents the aals, blue represents the hals

**Fig. 8:**
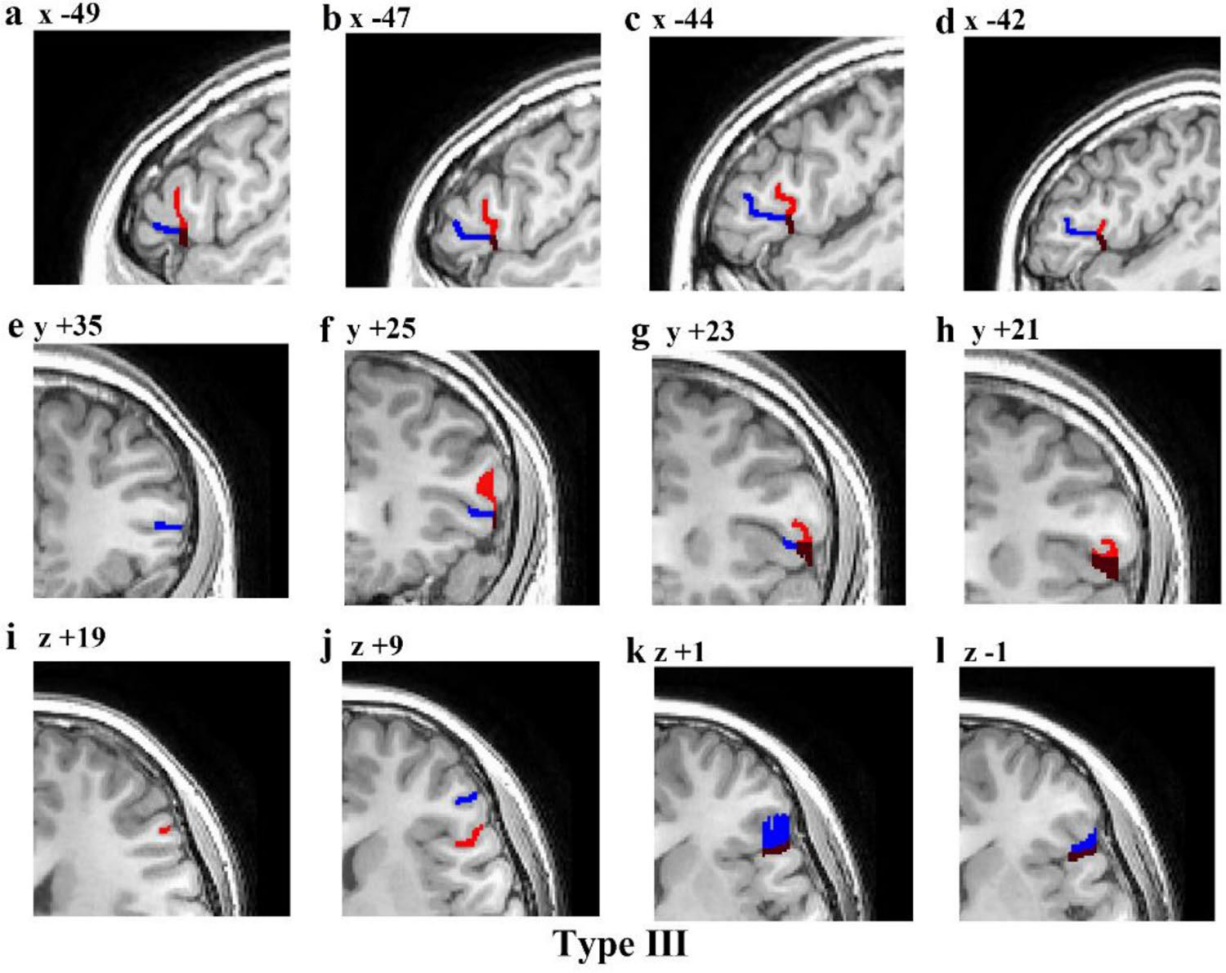
Example of an MRI scan (left hemisphere) illustrating the Type III morphology pattern. The first line is a series of sagittal sections (lateral to medial), with *x* coordinates indicated at the top of each section. The second line is a series of coronal sections (anterior to posterior), with *y* coordinates indicated at the top of each section. And the third line is a series of transverse sections (dorsal to ventral), with z coordinates indicated at the top of each section. Red represents the aals, blue represents the hals, brown represents the common stem of the aals and hals

**Fig. 9:**
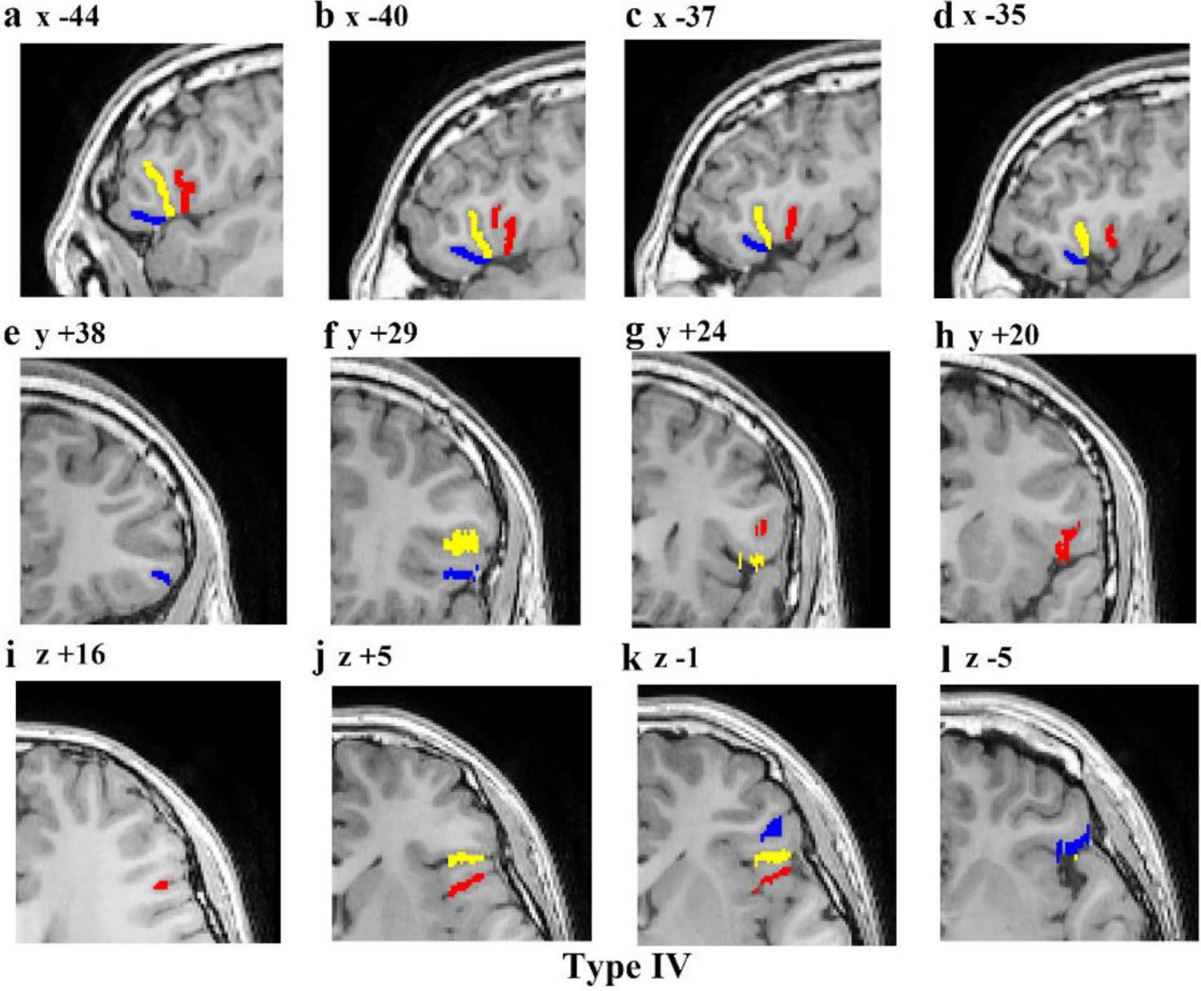
Example of an MRI scan (left hemisphere) illustrating the Type IV morphology pattern. The first line is a series of sagittal sections (lateral to medial), with *x* coordinates indicated at the top of each section. The second line is a series of coronal sections (anterior to posterior), with y coordinates indicated at the top of each section. And the third line is a series of transverse sections (dorsal to ventral), with *z* coordinates indicated at the top of each section. Red represents the aals, blue represents the hals, yellow represents the third anterior ramus of the lateral sulcus

The aals could be observed in 98.89% of hemispheres (98.89% of left hemispheres and right hemispheres). The hals could be observed in 98.33% of hemispheres (97.78% of left hemispheres and 98.89% of right hemispheres). Moreover, the morphological patterns of these two sulci could be categorized into four typical types (Fig. 5), the frequencies of which are presented in Table 1. Type I patterns could be identified in 55.56% of hemispheres (47.78% of the left hemispheres evaluated and 63.33% of the right hemispheres evaluated). The characteristics of this type of pattern were that the aals was clearly separated from the hals on the surface of the brain by a gyral bridge, forming the shape of the letter U (Fig. 5a–c). In 15 (six left and nine right) of these hemispheres, the aals and the hals formed the shape of the letter U on the surface of the brain but appeared to be connected in depth. In hemispheres with a Type II pattern, which was observed in 13.33% of hemispheres (16.67% of the left hemispheres evaluated and 10% of the right hemispheres evaluated), the aals and the hals sprang from a common point, forming the shape of the letter V (Fig. 5d–f). There were two instances in the left hemisphere in which the aals and the hals formed the shape of the letter V superficially but were separated in depth; this separation was clear when the subjects’ fundi were examined. In hemispheres with a Type III pattern, which was found in 12.78% of hemispheres (13.33% of the left hemispheres evaluated and 12.22% of the right hemispheres evaluated), the aals shared a common stem with the hals, forming the shape of the letter Y (Fig. 5g–i). There was a third anterior ramus of the lateral sulcus between the aals and the hals in regions with a Type IV morphological pattern (Fig. 5j–l). This type was found in 7.78% of hemispheres. Examples of MRI volumes illustrating each of the four main morphological patterns are provided in Figs. 6, 7, 8, and 9.

**Table 1:** Frequency of morphological patterns of the aals and the hals in the left and right hemispheres of the human brain

	Total no. LH	% 90 LH	Total no. RH	% 90 RH	Total no. LH + RH	%180 LH + RH	Pearson Chi-square	*P* value
Type I	43	47.78	57	63.33	100	55.56	4.410 (3.803)	0.036 (0.051)
Type II	15	16.67	9	10.00	24	13.33	1.731 (1.202)	0.188 (0.273)
Type III	12	13.33	11	12.22	23	12.78	0.050 (0.000)	0.823 (1.000)
Type IV	7	7.78	7	7.78	14	7.78	0.000	1
Only aals	1	1.11	1	1.11	2	1.11	–	–
Only hals	2	2.22	1	1.11	3	1.67	–	–
Bifurcated aals	4	4.44	1	1.11	5	2.78	–	–
Bifurcated hals	6	6.67	3	3.33	9	5	–	–

The values shown in brackets are the adjusted values after applying a continuity correction. Except for the four typical morphological patterns, the number of cases of other types is too small to be analyzed statistically. *aals* anterior ascending ramus of the lateral sulcus, *hals* horizontal ascending ramus of the lateral sulcus

**Fig. 10:**
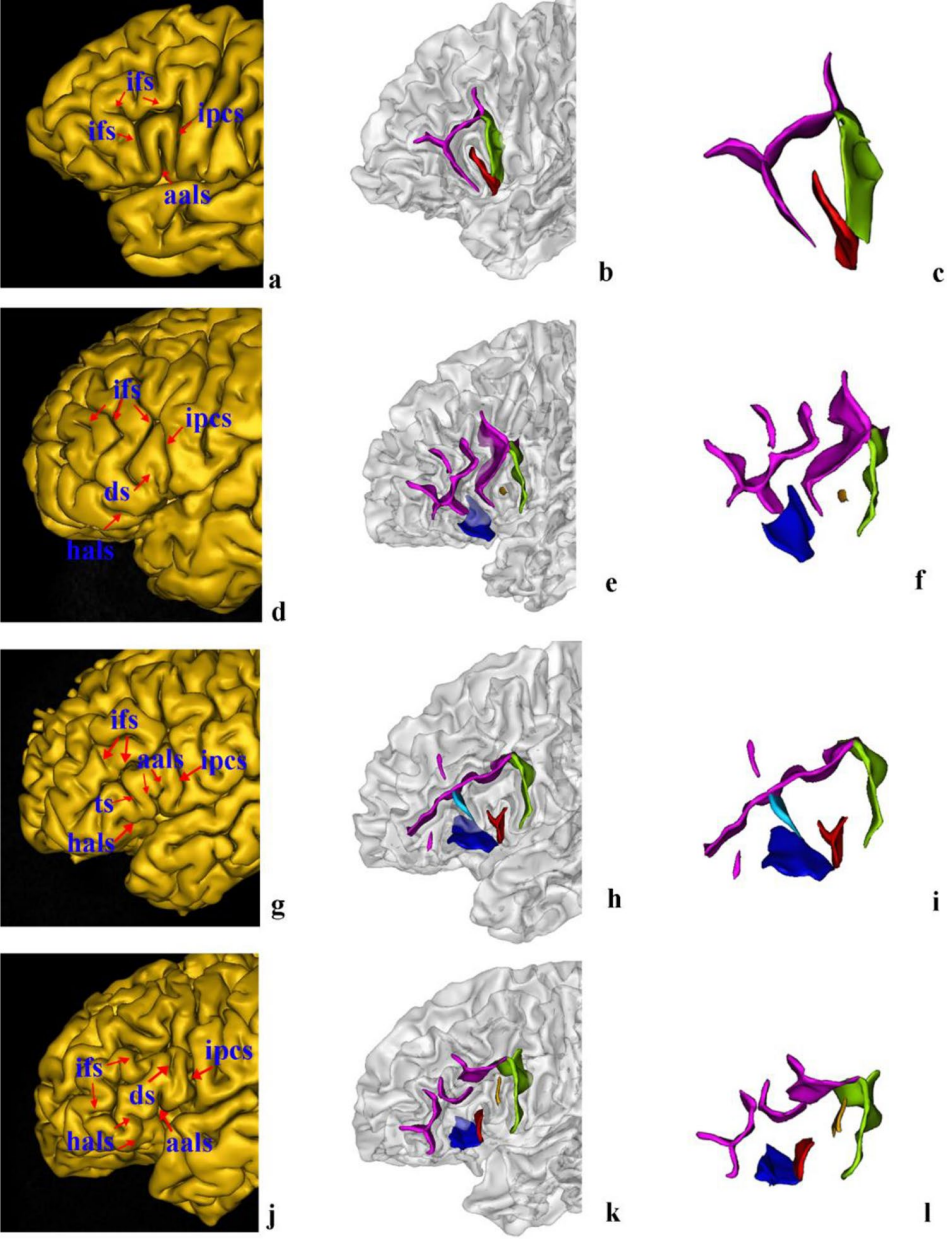
Additional examples of morphological patterns formed by the anterior ascending ramus of the lateral sulcus (aals) and the horizontal ascending ramus of the lateral sulcus (hals). Each example is illustrated by snapshot of surface extraction and sulci detail. **a**–**c** the hals was absent; **d**–**f** the aals was absent; **g**–**i** the aals is bifurcated at its dorsal end; **j**–**l** the hals is bifurcated at its anterior end

Additional morphological patterns were identified with less frequency (Fig. 10): (1) In 1.11% of hemispheres (1.11% of the left hemispheres evaluated and 1.11% of the right hemispheres evaluated), the hals was absent (Fig. 10a–c). (2) A lack of aals was observed in two (2.22%) of the left hemispheres and one (1.11%) of the right hemispheres examined (Fig. 10d–f). (3) The aals bifurcated at its dorsal end, which was found in four (4.44%) of the left hemispheres and one (1.11%) of the right hemispheres evaluated (Fig. 10g–i). (4) The hals bifurcated at its anterior end, which was found in six (6.67%) of the left hemispheres and three (3.33%) of the right hemispheres (Fig. 10j–l).

### Inter-hemispheric differences

There was a statistically significant difference (χ^2^ = 4.410, *p* < 0.05) between the left and right hemispheres in the Type I morphological pattern. However, this difference was no longer significant when a more conservative *p* value was used in a continuity correction, which is required for two-by-two contingency tables. Table 1 shows that Type I appeared more frequently in the right hemisphere than in the left hemisphere. Notably, no statistically significant difference was found between the left and right hemispheres in comparisons among hemispheres with Type II, Type III and Type IV morphological patterns. The results are shown in Table 1.

### Sex differences

For comparison of each of the four main morphology patterns, there was no statistically significant sex difference, as shown in Table 2.

**Table 2:** Sex difference analysis of four typical morphological patterns of the aals and the hals

Side		Sex		Pearson Chi-square	*p* value
		Male	Female		
Type I	L	27	16	1.230 (0.821)	0.267 (0.365)
	R	30	28		
Type II	L	7	8	2.240 (1.143)	0.134 (0.285)
	R	7	2		
Type III	L	6	6	0.434 (0.057)	0.510 (0.812)
	R	7	4		
Type IV	L	5	2	1.167 (0.292)	0.280 (0.589)
	R	3	4		

*L* left hemisphere, *R* right hemisphere

### Computation of sulci parameters

#### Inter-hemispheric differences

As shown in Tables 3 and 4, there was a statistically significant interhemispheric difference in the IL of the aals (*p* < 0.05). Compared to the left hemisphere, the inner length of the aals in the right hemisphere was longer.

**Table 3:** Descriptive statistics and results of two-way ANOVA for sulcal parameters of aals

Subgroup	Side	Sex		Hemisphere		Sex		Hemisphere $\times$ sex	
		Male	Female	F	P	F	P	F	P
AW (mm)	L	3.635 ± 1.198	3.715 ± ]0.836	0.010	0.919	0.122	0.728	0.008	0.931
	R	3.670 ± 1.454	3.718 ± 1.454						
OL (mm)	L	20.811 ± 7.460	18.890 ± 6.771	0.780	0.378	3.247	0.073	0.007	0.932
	R	21.217 ± 7.267	19.383 ± 6.055						
IL (mm)	L	23.937 ± 5.846	21.458 ± 4.629	5.646	0.019*	8.320	0.004*	0.002	0.964
	R	26.052 ± 6.620	23.493 ± 5.194						
AD (mm)	L	12.430 ± 3.022	10.505 ± 2.885	0.349	0.555	19.765	0.000*	0.000	0.995
	R	12.688 ± 2.980	10.759 ± 2.980						

The descriptive values are shown in mean ± SD

*AW* average width, *OL* outer length, *IL* inner length, *AD* average depth, *L* left hemisphere, *R* right hemisphere

Asterisk (*) indicates that *p* < 0.05

**Table 4:** Descriptive statistics and results of two-way ANOVA for sulcal parameters of hals

Subgroup	Side	Sex		Hemisphere		Sex		Hemisphere $\times$ Sex	
		Male	Female	F	P	F	P	F	P
AW (mm)	L	3.773 ± 2.027	3.302 ± 0.648	0.099	0.753	1.857	0.175	0.899	0.344
	R	3.605 ± 1.081	3.559 ± 0.511						
OL (mm)	L	14.832 ± 4.209	13.899 ± 5.447	0.008	0.928	7.896	0.006 *	3.400	0.067
	R	16.701 ± 8.421	12.205 ± 3.762						
IL (mm) AD (mm)	L	23.184 ± 3.9137	23.387 ± 7.169	0.289	0.592	2.065	0.153	2.847	0.094
	R	24.116 ± 4.400	21.585 ± 3.346						
	L	12.416 ± 2.328	11.583 ± 2.277	0.510	0.476	1.361	0.245	1.265	0.263
	R	11.748 ± 2.168	11.732 ± 1.948						

The descriptive values are shown in mean ± SD

#### Sex differences

The results of sex differences in sulcal parameters are also shown in Tables 3 and 4. Compared to females, the AD of males was deeper for the aals (*p* < 0.05). There was a significant sex difference in the IL of the aals (*p* < 0.05). Furthermore, there was a significant sex difference in the OL of the hals (*p* < 0.05). Males had greater IL of the aals and OL of the hals than females.

In addition, two-way ANOVA for sulci parameters showed no significant effect of the interaction between sex and hemisphere.

## Discussion

The present study determined the morphological variability of the anterior ascending ramus and the horizontal ascending ramus of the lateral sulcus. In our study, the morphological patterns of these two sulci were classified into typical and atypical types. The sulcal parameters were used to analyze hemispheric and sex differences in these two sulci.

The aals and hals are two defining sulci of the posterior ventrolateral frontal cortex of the human brain. These two sulci are well distinguished from other adjacent sulci considering the characteristics of the cortical surface and the depth of these two sulci. The aals could be observed in 98.89% of the hemispheres evaluated in our study, and the hals could be observed in 98.33% of the hemispheres examined in our study. The results were slightly different from other studies (Keller et al. 2007, 2009; Eser Ocak and Kocaeli 2017). Keller et al. (2007) found that the aals could be identified in 99% of all hemispheres examined, and the hals was identified in 98% of all hemispheres examined. An anatomic study of the morphology of Broca’s area reported that the frequency of the aals was 99%, and the hals was identified in 93% of the hemispheres examined (Eser Ocak and Kocaeli 2017). The discrepancy may be attributed to the great heterogeneity of the brain samples and the differences in sample sizes. The typical morphological patterns of these two sulci were classified into four types. Type I was the typical morphology, in which the aals and hals were clearly separated superficially. A number of studies have demonstrated this type of pattern (Keller et al. 2007; Sprung-Much and Petrides 2020). Interestingly, in our study, some hemispheres with a Type I pattern showed that the aals and the hals formed the shape of the letter U on the lateral surface but formed the shape of the letter V in depth. The literature on this finding is still scarce. In this study, BrainVISA was used to make the cerebral cortex transparent so that this type could be seen clearly. The aals and hals formed the shape of the letter V in hemispheres with a Type II pattern. In hemispheres with a Type III pattern, the aals shared a common trunk with the hals. The common trunk was either superficial or extended to the level of the insula. This finding was consistent with earlier studies that examined the morphology and spatial probability maps of the horizontal ascending ramus of the lateral fissure (Sprung-Much and Petrides 2020). There were cases in which a third anterior ramus of the lateral sulcus was located between the aals and the hals. This type refers to Type IV. This type of pattern was observed in 16% of the hemispheres evaluated in a previous study (Sprung-Much and Petrides 2020). Unlike Sprung-Much and Petrides’ results, the present study only found this pattern in 7.78% of cases. We speculate that this discrepancy could be due to the potential differences between Chinese and Western brains. In the present investigation, the aals was observed to bifurcate at its dorsal end but the hals bifurcates anteriorly. The results of Sprung-Much and Petrides’ study showed only that the hals bifurcates at its anterior end (Sprung-Much and Petrides 2020). This result may have been caused by the discrepancy between our classification standards and those of Sprung-Much and Petrides (2020), or it may have been due to our larger sample size. Overall, the morphology of these two sulci is complex and characterized by high intersubject variability. The results presented herein, with an analysis of the individual MRI volumes, provided a detailed way to distinguish the aals and hals.

In our study, interhemispheric differences in the frequency of Type I, Type II, Type III, and Type IV morphological patterns were analyzed. As shown in Table 1, none of the comparisons revealed significant differences. The results were similar to those described by Sprung-Much and Petrides (Sprung-Much and Petrides 2020). Although there was no hemispheric difference in the morphological types of the two sulci, there was hemispheric difference in the morphometric parameters of these two sulci. The sulcal parameters measured can reflect the quantitative information of sulci. The IL of the aals in the right hemisphere was longer than that in the left hemisphere and this difference was statistically significant, which may account for the higher global sulcal indices (g-SIs) found in the right hemisphere. The g-SI for each hemisphere is measured as the ratio between the total sulcal area and the outer cortex area, which reflects the complexity of sulcal folds (Liu et al. 2010). A study by Liu et al. (2010), which used automated methods to explore the g-SIs of both cerebral hemispheres in elderly individuals, found a higher g-SI in the right hemisphere than in the left hemisphere (Liu et al. 2010). There is no definite evidence of the association between the IL of the aals and language function, so the relationship between the hemispheric difference in IL of the aals and language function remains to be studied.

Our findings show that sex differences in the four typical morphological patterns of the aals and the hals were not significantly different, which was consistent with the study about sulcal morphology and volume of Broca’s area linked to handedness and sex (Powell et al. 2012). At present, the literature on sex differences in the morphological patterns of the aals and hals is scarce, especially in the Chinese population. This scarcity of information may be due to limited research methods and insufficient sample size. The AD of the aals showed significant sex differences. Males exhibited deeper AD of the aals than females. Sulcal depth of all sulci more strongly linked the volumes of adjacent gyri than global volumes (Van Essen 1997). Our study found that females were characterized by shorter IL of the aals, OL of the hals. These results may indirectly confirm women's advantages in relation to language, particularly in terms of fluency and verbal memory tasks (Mohammadi et al. 2017; Xu et al. 2020). This relationship between structure and function needs to be verified by further experiments. However, the results support previous studies reporting sex differences in brain structure and function (Cosgrove et al. 2007; Frere et al. 2020).

## Limitation and future directions

Several potential limitations existed in our study. The sample size was a limitation of this study, and neuroimaging research often requires a large amount of data. Although the sulci could be automatically reconstructed by BrainVISA software, we only studied 90 individuals due to the large amount of work required to manually trace the characteristics of individual MRI volumes of these two sulci in the present study. Hence, it is crucial to design a more convenient method to study the characteristics of individual MRI volumes of the sulcus. We used continuous correction methods to solve the potential issue of small effect sizes causing a lack of statistically significant differences in this study. Another limitation of our study is that the results should be verified by multiple methods. Although the accuracy of the results was analyzed by two methods of 3D reconstruction and individual MRI volume analysis, we should use a multimodal method in future research to better verify the results. In the next step, we will increase the quantity of data evaluated and use a multimodal method to strengthen our results. Moreover, we will add functional experiments to verify the relationship between brain structure and function.

In conclusion, the present study provides a detailed description of the morphology of the aals and the hals. The morphology of these two sulci presented here could aid in improving sulcal identification; moreover, the present study reported a detailed analysis of differences in quantitative information regarding sulci. The study sheds light on the characteristics of these two sulci, and it also provides guidance for the selection of a surgical approach in the treatment of this area. Moreover, the results presented here provide an anatomic basis for exploring the functions of Broca’s area.

## Data Availability

The data that support the findings of this study are available on reasonable request from the corresponding author.
